# Prerequisites for a dry powder inhaler for children with cystic fibrosis

**DOI:** 10.1371/journal.pone.0183130

**Published:** 2017-08-11

**Authors:** Anne J. Lexmond, Paul Hagedoorn, Henderik W. Frijlink, Bart L. Rottier, Anne H. de Boer

**Affiliations:** 1 Department of Pharmaceutical Technology and Biopharmacy, University of Groningen, Groningen, The Netherlands; 2 Division of Pediatric Pulmonology and Pediatric Allergology, Beatrix Children’s Hospital, University Medical Center Groningen, University of Groningen, Groningen, The Netherlands; 3 Groningen Research Institute for Asthma and COPD, University Medical Center Groningen, University of Groningen, Groningen, The Netherlands; Telethon Institute for Child Health Research, AUSTRALIA

## Abstract

Correct inhalation technique is essential for effective use of dry powder inhalers (DPIs), as their effectiveness largely depends on the patient’s inhalation manoeuvre. Children are an especially challenging target population for DPI development due to the large variability in understanding and inspiratory capacities. We previously performed a study in which we determined the prerequisites for a paediatric DPI in a mostly healthy paediatric population, for which we used an empty test inhaler with variable internal airflow resistance and mouthpiece. In the current study we investigated what specifications are required for a DPI for children with cystic fibrosis (CF), for which we expanded on our previous findings. We recorded flow profiles of 35 children with CF (aged 4.7–14.7 years) at three airflow resistances (0.031–0.045 kPa^0.5^.min.L^-1^) from which various inspiratory parameters were computed. Obstructions in the mouth during inhalation were recorded with a sinuscope. All children were able to perform a correct inhalation manoeuvre, although video analysis showed that children did not place the inhaler correctly in the mouth in 17% of the cases. No effect was found of medium to high airflow resistance on total inhaled volume, which implies that the whole resistance range tested is suitable for children with CF aged 4–14 years. No effect could be established of either mouthpiece design or airflow resistance on the occurrence of obstructions in the mouth cavity. This study confirms our previous conclusion that the development of DPIs specifically for children is highly desired. Such a paediatric DPI should function well at 0.5 L inhaled volume and a peak inspiratory flow rate of 20 to 30 L/min, depending on the internal airflow resistance. This resistance can be increased up to 0.045 kPa^0.5^.min.L^-1^ (medium-high) to reduce oropharyngeal deposition. A higher resistance may be less favourable due to its compromising effect on PIF and thereby on the energy available for powder dispersion.

## Introduction

Correct inhalation technique is essential for effective use of dry powder inhalers (DPIs), as the therapeutic effect of their use largely depends on the inhalation manoeuvre of the patient. The inhaled flow rate provides the energy for effective dispersion of the powder formulation and the delivery of a high fine particle dose, whereas the entire inhalation manoeuvre determines the lung deposition [1]. Therefore, sufficient inspiratory capacity is needed. In addition, understanding of how to perform the optimal manoeuvre is essential, which includes deep exhalation prior to and a sufficiently long breath hold after inhalation of the powder aerosol [2] to allow for deep penetration in the airways and time for sedimentation respectively. Both aspects are affected by patient characteristics like age and severity of disease [3–7]. It is, therefore, important to study the prerequisites for correct use of DPIs in selected patient populations.

Children constitute a special and highly heterogeneous target population for inhaled drug delivery. Lung physiology and cognitive abilities are in development and even within the same age group a large variability in understanding and inspiratory capacity may exist between individual children. Therefore, serving this target population is highly challenging and only devices that perform largely independent of the patient may be suitable for this group. Young children may be unable to understand and comply with complex inhalation instructions, whereas the immature inspiratory capacity may result in the inhalation of only small volumes at low flow rates. Designers of DPIs for this patient group should take account of these limitations and variability.

Many children with cystic fibrosis (CF) are treated with inhaled antibiotics, which are frequently administered by nebulisation. To reduce the burden of antibiotic nebulisation, the first generation capsule-based DPIs with antibiotics (tobramycin and colistimethate) is currently used, but these DPIs are registered for patients with CF from the age of 6 years onward. They require inhaled volumes ≥1 L at a moderate to high flow rate (≥40 L/min) to deliver the entire dose in the capsule in one inhalation [8]. To ensure complete dose emission, patients are therefore advised to inhale multiple times from one capsule if necessary [9,10]. One dose dry powder tobramycin is contained in four capsules that have to be inhaled consecutively, quadrupling the number of inhalations per dose administration. Young children cannot achieve such high volumes and high flow rates [7], and thus they require multiple inhalations to receive the full dose. This makes the administration lengthier and more burdensome, especially for tobramycin. Additionally and perhaps more importantly, most studies report only the effects of flow rate and inhaled volume on the emitted dose [8,11], while the dispersion efficacy may be severely compromised when the device is operated at lower values. It has been shown for the colistimethate capsule DPI that dispersion is already poor when operated at a flow rate of 65 L/min (4 kPa), resulting in a fine particle fraction of 1–5 μm of 20% of the emitted dose [12]. At this flow rate, an estimated 40 to 50% of this fraction is deposited in (adult) lungs [13], resulting in an effective lung dose of around 10% of the emitted dose. Larger particles (>5 μm) are more likely to be deposited in the oropharyngeal region [13]. When the patient inhales with a lower flow rate, less energy is available for dispersion and a shift towards larger particles (agglomerates) and hence more oropharyngeal deposition will occur. In children the effects of this reduced dispersion efficacy on oropharyngeal deposition can be even more detrimental due to their smaller anatomical dimensions. Therefore, the development of DPIs especially for children that are designed to function optimally at low inhaled flow rates and volumes is warranted. However, it is not known what specifications are required for a DPI for children with CF.

**Fig 1:**
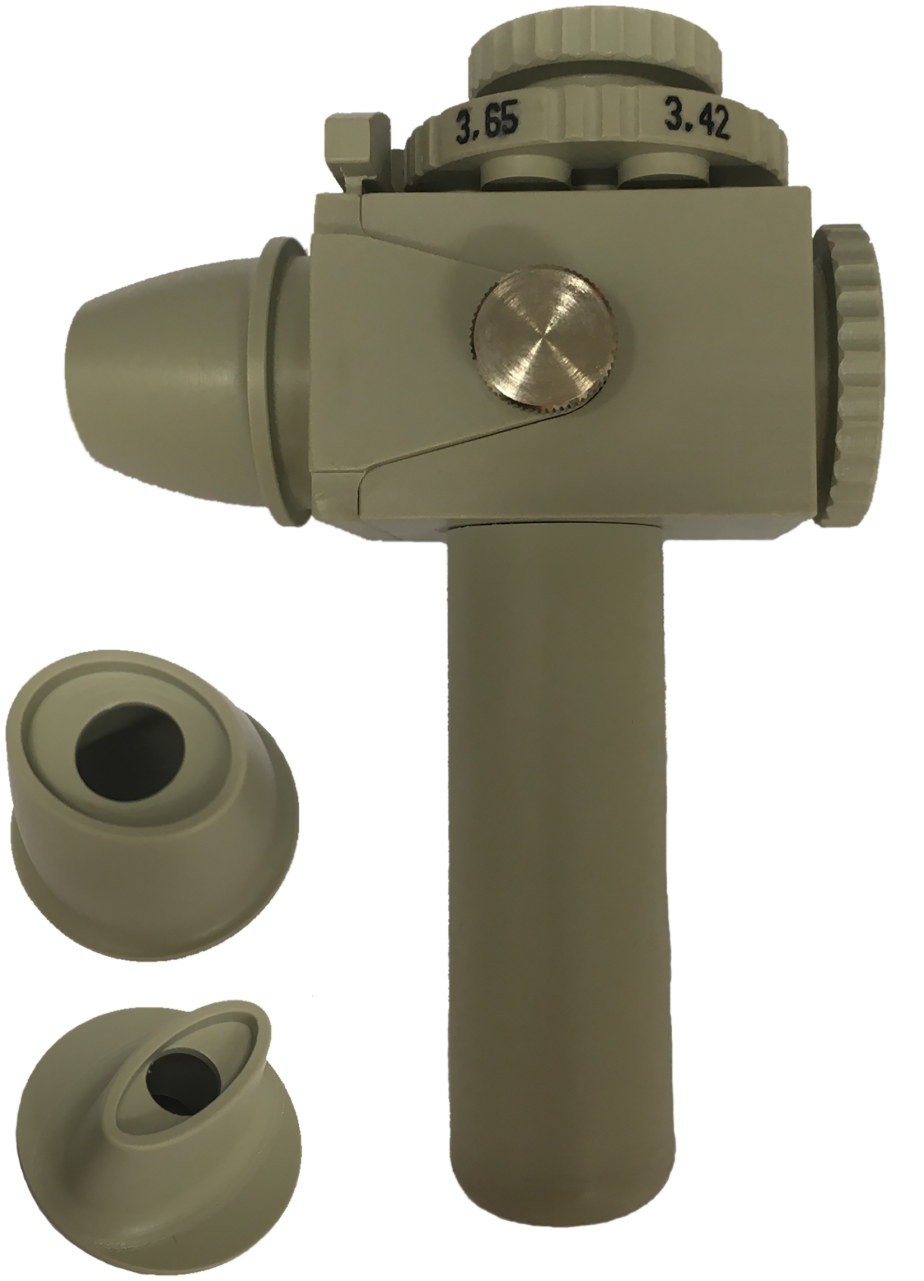
The test inhaler used in this study with the oval (top) and oblong mouthpiece design. The test inhaler is equipped with a differential pressure gauge through the bottom of the handle and connected to a data acquisition system for recording of the inspiratory flow manoeuvres. The rotatable ring with orifices of different sizes on top is used to set the airflow resistance. The test inhaler has been described previously in more detail [7].

Previously we conducted a study in healthy school children to investigate their cognitive and inspiratory capacities regarding dry powder inhalation [7]. In this study, we determined requirements for a paediatric DPI using an instrumented test inhaler with variable internal resistance against airflow and mouthpiece design (Fig 1). Our primary aim was to assess from what age on children have the cognitive capacity to operate a DPI correctly, how their inspiratory capacities were and how various aspects of the inhalation manoeuvre are affected by the inhaler’s internal airflow resistance and its mouthpiece design. We used the data to determine the optimal design variables for the development of a paediatric DPI and found that such a DPI should deliver a sufficiently high fine particle dose within an inhaled volume of 0.5 L, at a peak inspiratory flow rate of no more than 25–40 L/min (depending on the airflow resistance). However, the participants in this study were mostly healthy. Chronic pulmonary infection is a hallmark of lung disease in CF and the resulting inflammatory changes of the airway may result in reduced lung function. Therefore, we conducted this study in children with CF for which we used the design variables that appeared to suit healthy children best. Although children with CF may have reduced lung function already at a young age [14], they often still demonstrate normal spirometry, which deteriorates significantly as they get older [15]. Therefore, we expected that the youngest children with CF would have an inspiratory capacity that corresponds fairly well to that of healthy children, but that the older children would lag behind their peers. We investigated whether the physical limitations for DPI use found in young healthy children also apply to older children with CF.

## Subjects and methods

### Study population

Participants were recruited in the outpatient clinic of the Beatrix Children’s Hospital in Groningen, the Netherlands. Children with CF in the age range 4–14 years of age were eligible for participation. Written informed consent from the parent(s)/guardian(s)—and if applicable, from the children themselves—was obtained. The study was conducted according to the principles expressed in the Declaration of Helsinki and approved by the Medical Ethics Committee of the University Medical Center Groningen. Tests were conducted prior to the routine check-up visit that children with CF undergo every three months in the Netherlands.

### Study design

The study was an exploratory non-therapeutic observational study in which the applicability of dry powder inhalation in children with CF was investigated with an empty instrumented test inhaler described previously [7]. A computer with data acquisition software (LabView, National Instruments BV, The Netherlands) recorded the flow profiles (flow rate, derived from the pressure drop across the inhaler, as function of time) of the children’s inhalations through the inhaler. The internal airflow resistances and mouthpiece designs of this test inhaler can be varied and the ones used in this study were those found to be most suitable for children in the previous study with healthy children. The airflow resistances were 0.031 (R4), 0.039 (R3), and 0.045 kPa^0.5^.min.L^-1^ (R2), covering the range of medium to high airflow resistance according to the definition of the ERS/ISAM task force [16]. The two mouthpiece designs used were oblong and oval (Fig 1). The oblong mouthpiece was most preferred by the healthy children in the previous study, whereas the oval mouthpiece appeared to have greatest positive effect on the passageway for an aerosol through the mouth cavity [7].

The study comprised two randomised procedures (see for the randomisation the next paragraph). Firstly, flow profiles of three inhalations were recorded per child. Secondly, each child performed three additional inhalations during which the effects of mouthpiece design and internal airflow resistance on the passageway for the aerosol through the mouth cavity were investigated using a sinuscope (Olympus WA96200A, Olympus Winter & Ibe GmbH, Germany) that was placed inside the test inhaler. The video recordings from the sinuscope were used to observe whether or not an obstruction (e.g. from tongue, teeth or cheeks) existed in the aerosol passageway towards the throat during the inhalation manoeuvre. These videos were evaluated qualitatively by an experienced investigator, following earlier experience that the observations of two independent investigators were highly similar [7]. Obstructions were defined as less than a third (by estimation) of the throat visible, a raised tongue, cheeks curved inward, or lips and/or teeth in front of the lens. It was also investigated whether condensation of damp air on the lens of the sinuscope had occurred, which may be an indication of exhalation into the inhaler.

Randomisation for the procedures was performed prior to the measurements by computer (Excel, Microsoft). One of the two mouthpieces was randomly assigned for the entire first procedure and the order in which the three airflow resistances were tested was randomised as well by computer. For the second procedure, two mouthpiece designs and two resistances were randomly tested per child.

Each child received the following instructions for the inhalation manoeuvre:

- Sit or stand up straight;
- Keep the inhaler in upright position;
- Exhale completely, away from the inhaler;
- Place the inhaler in the mouth and seal teeth and lips around it;
- Inhale as forcefully and as long as possible;
- Take the inhaler from the mouth;
- Hold your breath for ten seconds (or as long as comfortable);
- Exhale.

The children were asked to repeat the instructions or demonstrate the manoeuvre and when they did so correctly, the measurements commenced.

The inspiratory parameters that were calculated were the maximum pressure drop (dP_max_), the peak inspiratory flow rate (PIF), the mean inspiratory flow rate (MIF), the flow increase rate (mean acceleration in flow rate from 20% to 80% of PIF; FIR_20-80%_), the inhaled volume (V_i_), and the total inhalation time (t_i_).

### Data analysis

Routine descriptive statistics of the subject characteristics were performed. Exploratory data analysis was performed to identify data distribution trends and normality was tested using Shapiro-Wilk normality test. Because the sample size was limited and the population was quite skewed towards older children (twelve children ≥12 years of age), variables were generally not normally distributed. Therefore, for all variables Spearman’s correlation coefficients for non-normally distributed variables were calculated to study the correlation between the inspiratory parameters and age. Fixed effects linear mixed models were then used to estimate the effects of airflow resistance and the children’s characteristics inspiratory parameters after log transformation to normalise the distributions. The presence of obstructions in the oral cavity and the effects of mouthpiece design and airflow resistance thereon were evaluated qualitatively. Analyses were performed with SPSS 23 (IBM) and Prism 5.0 (GraphPad Software). All statistical testing was two-sided, with an α of 0.05.

## Results

### Study population

35 children were included in the study. All of them were able to comprehend the instructions and to finish the first procedure. One subject (age 4.9 years) did not perform the second procedure, because she was tired after the first and lost motivation for completing the test. Therefore, the investigator and child’s mother agreed that it was better to end the measurements. Descriptive statistics of the study population are shown in Table 1.

**Table 1:** Descriptive statistics of the study subjects.

	Mean	Median	SD	Range
Age (years)	9.9	9.1	3.4	4.7–14.7
Height (cm)	141.7	139.0	19.5	108.3–179.6
Weight (kg)	35.4	30.6	13.0	18.5–63.8
FVC (L)	2.40	2.06	1.09	0.70–6.00
FVC (%pred)*	98	96	18	44–136
FEV_1_ (L)	1.92	1.73	0.78	0.41–4.26
FEV_1_ (%pred)*	93	93	20	30–132
FEV_1_%FVC	81	81	8.3	58–97
Sex	Male: 18 (51%)		Female: 17 (49%)	

FEV_1_: forced expiratory volume in one second; FVC: forced vital capacity.

*FEV_1_ and FVC as %predicted were based on Zapletal reference values [17].

### Flow profiles and inspiratory parameters

Of a total of 105 recorded flow profiles, six were excluded. One subject was mentally not feeling well and did not put any effort into the inhalations. For that reason these flow profiles were excluded from analysis. One flow profile of a second subject was excluded because the inhalation was interrupted after 0.5 s. Two flow profiles of two more subjects were excluded due to technical errors.

**Fig 2:**
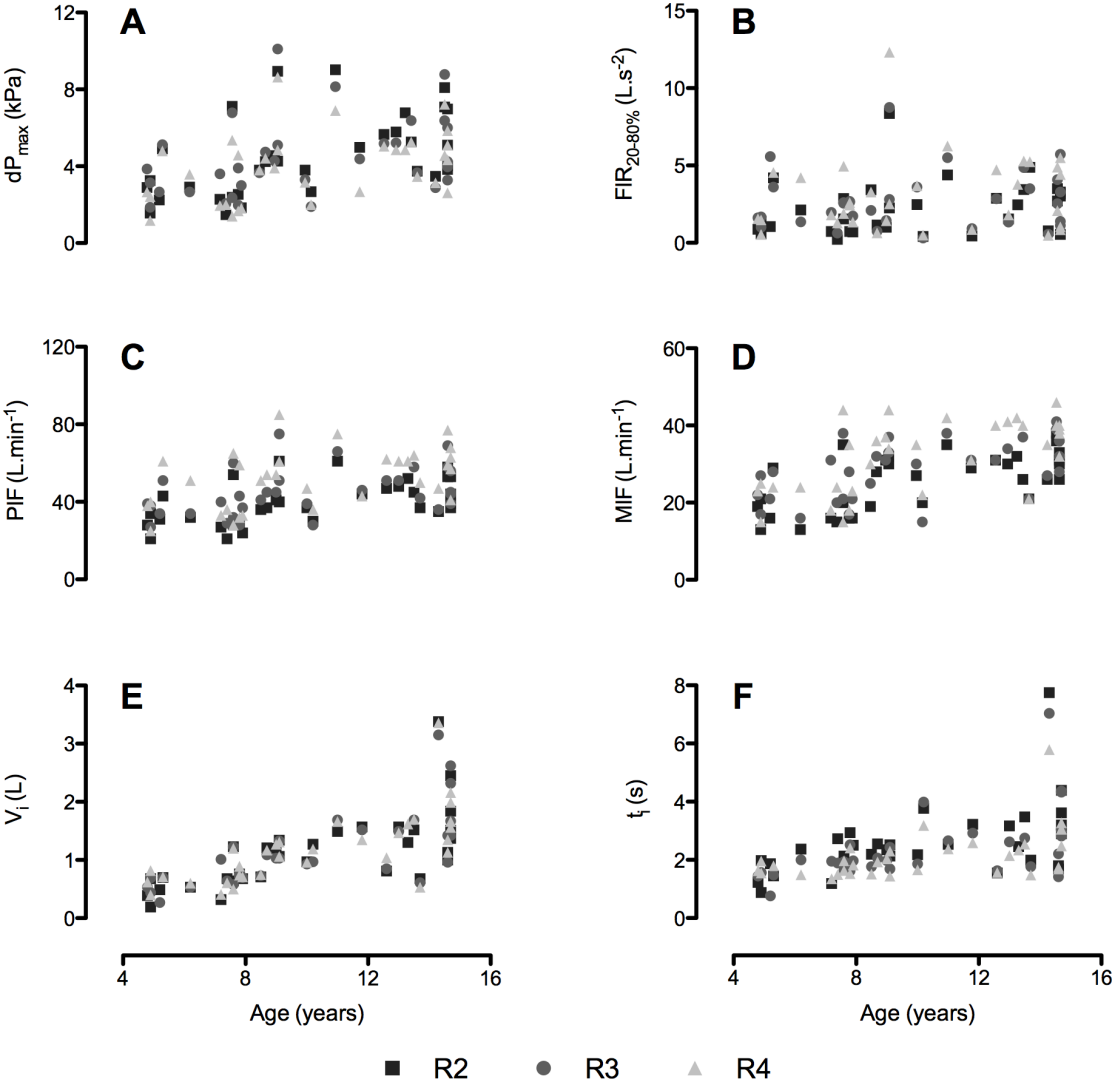
Scatter plots of the inspiratory data versus age. A) maximal pressure drop (dP_max_); B) flow increase rate (FIR_20-80%_); C) peak inspiratory flow rate (PIF); D) mean inspiratory flow rate (MIF); E) inhaled volume (V_i_); F) inhalation time (t_i_). The dataset can be found in the Supporting Information (S1 File).

Scatter plots of the inspiratory parameters versus age are presented in Fig 2. All parameters except for FIR_20-80%_ were significantly positively correlated with age. Two children (4.9 and 5.2 years old) inhaled 0.42 L and 0.38 L respectively, all other children reached values of 0.50 L or higher. Three children (4.9, 7.4, and 7.9 years) did not reach a PIF of 25 L/min through the high resistance mode of our test inhaler (0.045 kPa^0.5^.min.L^-1^). The lowest PIF values on each resistance were from the same subject (4.9 years), with 21, 27, and 25 L/min at R2, R3, and R4 respectively.

### Data processing

The inspiratory parameters from children aged 4–14 years were modelled with the resistance modes, subject age, height, sex, and lung function parameters FVC (%pred) and FEV_1_%FVC as covariates (Table 2). Since height and age are strongly correlated (*r* = 0.95), a variable was included for the residual variation in height instead of height to prevent multicollinearity. For the same reason FEV_1_%FVC rather than FEV_1_ (%pred) was included, as the latter correlates more strongly with FVC (%pred).

**Table 2:** Linear mixed model parameter estimates of the effects of airflow resistance and the children’s characteristics and lung function on the inspiratory parameters.

	dP_max_ (kPa)		FIR_20-80%_ (L.s^-2^)		PIF (L.min^-1^)		MIF (L.min^-1^)		V_i_ (L)		t_i_ (s)	
	Parameter estimate ± SE	*p*-value	Parameter estimate ± SE	*p*-value	Parameter estimate ± SE	*p*-value	Parameter estimate ± SE	*p*-value	Parameter estimate ± SE	*p*-value	Parameter estimate ± SE	*p*-value
Intercept	.200 ± .457	.67	.672 ± .908	.47	1.51 ± .276	< .001	1.35 ± .259	< .001	-.212 ± .330	.53	.226 ± .306	.47
R2^a^	.061± .017	**.001**	-.143 ± .039	**<.001**	-.113 ± .010	**<.001**	-.092 ± .014	**<.001**	-.026 ± .018	.16	.068 ± .017	**<.001**
R3^a^	.063 ± .017	**<.001**	-.033 ± .039	.39	-.055 ± .010	**<.001**	-.036 ± .014	**.011**	-.005 ± .018	.80	.034 ± .017	.057
Sex^b^	.009 ± .060	.88	.021 ± .118	.86	.000 ± .036	1	-.013 ± .034	.71	-.082 ± .043	.069	-.070 ± .040	.091
Age (years)	.030 ± .010	**.004**	.012 ± .019	.54	.019 ± .006	**.004**	.023 ± .006	**<.001**	.050 ± .007	**<.001**	.028 ± .007	**<.001**
Height_resid_^c^ (cm)	-.002 ± .005	.62	-.005 ± .009	.60	-.001 ± .003	.64	-.004 ± .003	.18	.003 ± .003	.42	.006 ± .003	.057
FVC (%pred)	.005 ± .002	**.032**	.004 ± .004	.27	.003 ± .001	**.048**	.002 ± .001	.057	.004 ± .001	**.022**	.001 ± .001	.35
FEV_1_%FVC	-.005 ± .002	.27	-.011 ± .009	.24	-.003 ± .003	.27	-.004 ± .003	.14	-.007 ± .003	**.032**	-.004 ± .003	.24
Random intercept	.027 ± .008		.103 ± .030		.010 ± .003		.008 ± .002		.013 ± .004		.011 ± .003	
Residual	.005 ± .001		.024 ± .004		.002 ± .000		.003 ± .001		.005 ± .001		.005 ± .001	
*N*	99		99		99		99		99		99	

dP_max_: maximal pressure drop; FEV_1_: forced expiratory volume in one second; FIR_20-80%_: flow increase rate; FVC: forced vital capacity; MIF: mean inspiratory flow rate (over the entire curve); PIF: peak inspiratory flow rate; t_i_: inhalation time; V_i_: inhaled volume. Inspiratory parameter values were log-transformed prior to analysis. R2 = 0.045 kPa^0.5^.min.L^-1^; R3 = 0.039 kPa^0.5^.min.L^-1^.

^a^ Reference category is R4 (0.031 kPa^0.5^.min.L^-1^).

^b^ Reference category is male.

^c^ Height_resid_ = Height—Height_exp_. Height_exp_ obtained by linear regression: Height_exp_ = 5.46 * Age + 87.66 (*r* = .95).

The resistance modes affected all inspiratory parameters except inhaled volume. At higher resistance, dP_max_ and inhalation time increased, whereas FIR_20-80%_, PIF and MIF decreased. In the multivariable analysis, age significantly independently affected all parameters except FIR_20-80%_. Inhaled volume and inhalation time tended to be lower for girls than boys (*p* = 0.069 and *p* = 0.091 respectively). Another important covariate was FVC, which significantly positively affected dP_max_, PIF, and inhaled volume. FEV_1_%FVC on the other hand was found to negatively affect inhaled volume only. In Table 3 the estimated means of the inspiratory parameters are shown per resistance mode to illustrate the extent to which the parameters were affected by resistance in our study population.

**Table 3:** Estimated means of the inspiratory parameters per resistance mode.

	dP_max_ (kPa)		FIR_20-80%_ (L.s^-2^)		PIF (L.min^-1^)		MIF (L.min^-1^)		V_i_ (L)		t_i_ (s)	
	Mean	95% CI	Mean	95% CI	Mean	95% CI	Mean	95% CI	Mean	95% CI	Mean	95% CI
R2	4.04	[3.50–4.65]	1.63	[1.23–2.18]	38.2	[35.1–41.7]	24.4	[22.4–26.6]	0.95	[0.85–1.06]	2.33	[2.11–2.58]
R3	4.05	[3.52–4.67]	2.10	[1.58–2.80]	43.7	[40.1–47.6]	27.8	[25.5–30.2]	1.00	[0.89–1.11]	2.15	[1.95–2.38]
R4	3.50	[3.04–4.04]	2.27	[1.71–3.03]	49.7	[45.5–54.1]	30.1	[27.7–32.8]	1.01	[0.90–1.12]	2.00	[1.80–2.20]

dP_max_: maximal pressure drop; FEV_1_: forced expiratory volume in one second; FIR_20-80%_: flow increase rate; FVC: forced vital capacity; MIF: mean inspiratory flow rate; PIF: peak inspiratory flow rate; t_i_: inhalation time; V_i_: inhaled volume. Covariates in the model are evaluated at: Age = 10.0 years; H_resid_ = -0.004 cm; FVC = 99.5%pred; FEV_1_%FVC = 81.8. Values obtained by naïve back transformation of log-transformed estimates.

### Recordings of the mouth cavity during inhalation

In total 102 videos were recorded. In 34% of the video recordings, the passageway was wide open without visual obstructions. In the 66% of partly obstructed passageways it was mostly the tongue that prevented a clear view on the back of the mouth cavity. In 17 videos (17%) of nine children teeth and/or lips were visible (for three children one out of three, for three children two out of three, and for three children three out of three recordings). Condensation of air on the sinuscope lens (an indication of exhalation into the inhaler) occurred in 30% of all recordings. A rank order was assigned to the three videos per child to investigate whether the variables airflow resistance and mouthpiece design had an effect on the incidence of obstructions. Table 4 shows the percentages in which a resistance mode or mouthpiece design had a positive effect on the passageway relative to the comparator. Because of the qualitative assessment and the small number of cases, no significant effects of any of these variables can be concluded and the results shown are only indicative.

**Table 4:** Percentages of cases in which a DPI design variable affected the aerosol passageway positively relative to the comparator design variable.

Resistance*		Mouthpiece	
R2	41%	Oval	38%
R3	12%	Oblong	30%
R4	26%	No difference	32%
No difference	21%		

The dataset can be found in the Supporting Information (S2 File).

*Percentages per resistance mode are corrected for the percentage of cases in which each resistance mode was used.

## Discussion

This study shows that after good instruction, even young children with CF are able to understand how to inhale correctly through a DPI. Our findings clearly demonstrate that there is no effect of medium to high airflow resistance on total inhaled volume, which implies that the whole resistance range tested is suitable for children with CF aged 4–14 years. No effect could be established of either mouthpiece design or airflow resistance in the tested range on the occurrence of obstructions in the mouth cavity.

Successful use of a DPI requires that a patient understands how to operate the device and is capable of handling the inhaler adequately and performing the correct inhalation manoeuvre. Children constitute a particularly challenging population for DPI use because of their cognitive and physical development as they grow up. We investigated whether children with CF are technically able to operate a DPI using a test inhaler. All children in our study, including the 4-year olds, demonstrated understanding of the inhalation manoeuvre, although one subject (7.6 years) subsequently failed to do comply with the instructions during the measurements. This was attributed to poor motivation rather than incomprehension, as she was mentally feeling unwell. Furthermore, correct use of an inhaler also includes correct placement of the inhaler in the mouth. Our observation of teeth and/or lips in front of the sinuscope lens in a significant number of cases shows the importance of checking whether the inhaler is actually placed correctly in the mouth prior to inhalation.

Most studied factors affect the airflow inhaled through the DPI, related to both device (internal resistance) and patient (age, sex, height, lung function). By modelling the inspiratory parameters with these covariables, we investigated whether they contribute significantly. In this study with children with CF, the resistance modes appeared to affect all inspiratory parameters except inhaled volume, which implies that the three resistance modes tested can be considered equally suitable for children with CF in the age range 4–14 years. These findings are in line with the findings of Tiddens et al., who studied the effect of inhaler resistance in the range 0.019–0.048 kPa^0.5^.min.L^-1^ on the inspiratory capacity of CF patients ≥6 years of age, and found no effect of airflow resistance on inhaled volume [5]. Higher internal airflow resistance results in a lower inspiratory flow rate, thereby reducing oropharyngeal deposition [13], for which reason a higher resistance may actually be preferred. However, oropharyngeal deposition can also be dramatically increased if the inhaler does not disperse well at low flow rates [18]. Therefore, the inhaler design must ensure effective dispersion at lower inspiratory effort to benefit from the effect of lower inspiratory flow rate on oropharyngeal deposition. In our study population the maximum pressure drop over the inhaler was significantly higher at a medium-high and high resistance compared to the medium resistance, which implies that more energy is available for dispersion of a drug powder, thus providing additional argument for a medium-high to high resistance inhaler.

Our results further show that a child’s physical characteristics are important determinants for its performance. Our dataset is not suitable for building a prediction model with these characteristics, due to the small sample size and an unequal distribution of children over the age range as a result of the exploratory study design. However, it can be assumed that besides a lower age and height, an impaired lung function also affects a child’s inspiratory performance negatively. This could be especially relevant for children suffering from an exacerbation of their disease. In particular low FVC (%pred) may be an indicator that a child will not be able to use a DPI as well as can be expected based on their age.

Key inspiratory parameters for optimal inhaler performance, and thus inhaler design, are PIF and inhaled volume. A sufficiently high PIF is required for fast and complete entrainment of the powder formulation from the dose compartment and effective dispersion of that formulation into a suitable aerosol for inhalation. A sufficiently large inhaled volume is needed for transport of the aerosolised drug particles into the lower airways. We previously concluded that a paediatric DPI should perform adequately at a PIF of 25–40 L/min (depending on the airflow resistance) and deliver a sufficient fine particle dose at an inhaled volume of 0.5 L [7]. These values, based on a study population of mostly healthy children of whom the youngest was 5.3 years old, are slightly lower than those from Tiddens et al. who studied CF patients and concluded that a DPI that performs optimally at a peak inspiratory flow rate of ≤ 30 L/min with and inhaled volume ≤ 1 L would be ideal for most CF patients ≥ 6 years of age with different severities of disease [5]. They argue that the need for a larger inhaled volume could partly be resolved by inhaling the total dose in multiple breaths [5], as is advised for the capsule-based antibiotic DPIs. This is of course a possibility, although there is likely a limit to the number of inhalations beyond which compliance becomes an issue. In the present study we had even younger children demonstrating a correct inhalation technique and all but two inhaled 0.5 L or more and all but three reached a PIF of 25 L/min through the high resistance mode of our test inhaler. This may suggest that although children younger than 5 years can be technically capable of performing the correct manoeuvre, their smaller inspiratory capacity may eventually restrict the feasibility of a breath-operated paediatric DPI for this age group, as accommodating to even lower values for the inspiratory parameters may compromise device efficacy beyond what is acceptable.

The presence of obstructions in the mouth cavity during inhalation appeared lower than in healthy children, where we found incidences of obstructions up to 90% [7]. The occurrence of moisture condensation from exhaled air on the sinuscope lens was comparable to that in healthy children (39%) [7] and thus, our previous recommendation to equip a paediatric DPI with suitable means to protect the inhaler’s interior against exhalation through the device is still relevant. In the present study we limited the design variables to those that appeared most suitable for children in the first study. We have found no further evidence that any of these design variables is more suited than the others and it appears that all three resistances and both mouthpiece designs can be used for a paediatric DPI.

Additional design aspects that should be considered are ease of dose preparation and inhaler robustness. Although it can be argued that such properties are important for any inhalation device, particularly for a paediatric DPI it would be valuable if only a few simple steps are required to prepare the inhaler for use, as well as a robust design that can withstand rough handling. This could have a positive effect on the child’s ability to use the inhaler independently.

In conclusion, we found no effect of airflow resistance in the range medium to high on inhaled volume, which implies that the whole range tested is suitable for children with CF aged 4–14 years, provided that the effects of airflow resistance on the other inspiratory parameters such as PIF are accounted for in the inhaler design and efficacy. We could not establish an effect of either mouthpiece design or airflow resistance on the occurrence of obstructions in the mouth cavity. The present study in CF patients confirms our previous study in healthy subjects and supports our conclusion that the development of DPIs specifically for children is highly desired. Prerequisites for a paediatric DPI that can be operated by the majority of CF patients ≥ 4 years of age are that it should function well at 0.5 L inhaled volume and a PIF of 20 to 30 L/min, depending on the internal airflow resistance. Airflow resistance can be increased up to 0.045 kPa^0.5^.min.L^-1^ (medium-high). A higher resistance may be less favourable due to its compromising effect on PIF and thereby on the energy available for powder dispersion.

## Supporting information

S1 File Datasheet of patient characteristics and flow profile data. (XLSX)

S2 File Datasheet of the video analysis. Qualititative assessment of the effects of resistance and mouthpiece design on obstructions in the mouth cavity upon inhalation.
(XLSX)
